# A Review on Osteopathic Manipulation in Patients With Headache

**DOI:** 10.7759/cureus.66242

**Published:** 2024-08-05

**Authors:** H V Sharath, Pavan Teja Nadipena, Moh'd Irshad Qureshi, Pratik Phansopkar

**Affiliations:** 1 Department of Pediatric Physiotherapy, Ravi Nair Physiotherapy College, Datta Meghe Institute of Higher Education and Research (Deemed to be University), Wardha, IND; 2 Department of Osteopathic Medicine, Dr. Hullumani's Polyclinic and Rehabilitation, Bangalore, IND; 3 Department of Neurophysiotherapy, Ravi Nair Physiotherapy College, Datta Meghe Institute of Higher Education and Research (Deemed to be University), Wardha, IND; 4 Depatment of Musculoskeletal Physiotherapy, Ravi Nair Physiotherapy College, Datta Meghe Institute of Higher Education and Research (Deemed to be University), Wardha, IND

**Keywords:** headache, musculoskeletal manipulation, exercise training, chronic headache and tension-type headache, osteopathy and rehabilitation

## Abstract

Headaches are a common neurological disorder, significantly impacting patients' quality of life. Traditional treatments include pharmacological and nonpharmacological approaches. Osteopathic manipulative treatment (OMT) is a holistic, hands-on technique used by osteopathic physicians to alleviate pain and improve function by addressing musculoskeletal dysfunctions. This review aims to evaluate the effectiveness of osteopathic manipulation in managing headaches, focusing on the different types of headaches, the specific techniques used, and the overall outcomes reported in clinical studies. A comprehensive literature search was conducted across multiple databases, including PubMed, Google Scholar, and MEDLINE, to identify relevant studies published in the past two decades. Inclusion criteria were studies involving adult patients diagnosed with headaches and treated with OMT. Both randomized controlled trials (RCTs) and observational studies were included. The review identified 15 studies meeting the inclusion criteria. Evidence suggests that OMT can be beneficial in reducing the frequency, intensity, and duration of headaches, particularly tension-type headaches (TTHs) and migraines. Techniques such as myofascial release, cranial osteopathy, and muscle energy techniques were commonly employed. Many studies reported significant improvements in patients' quality of life and functional status post-treatment. However, the heterogeneity in study designs, sample sizes, and outcome measures warrants cautious interpretation of the results. Osteopathic manipulation shows promise as a complementary approach for managing headaches, with positive effects on pain relief and functional improvement. Further large-scale, high-quality RCTs are needed to confirm these findings and to establish standardized treatment protocols. Integrating OMT into multidisciplinary headache management strategies could potentially enhance patient outcomes and reduce reliance on pharmacological interventions.

## Introduction and background

Most individuals suffer from headaches, which have a big impact on public health. As per the estimates of World Health Organization (WHO), 47% of individuals around the globe experience active headache problems, 10% with migraines, 38% with tension-type headaches (TTHs), and 3% with chronic headaches. The condition is one of the top 10 most incapacitating conditions [1]. Furthermore, the most common neurological complaint that patients report to neurologists and general practitioners is headache [2]. The majority of headache diseases are primary, with migraine and TTH being particularly significant once due to the population these affect, 60%-80% suffer from TTH, and 15% (5.6% of men and 18.3% of women) have migraines [3]. The most common type of secondary headache is cervicogenic headache (CGH); it occurs when any conditions impact the neck's muscles, intervertebral discs, and bone structure rather than the skull. Globally acknowledged as a unique clinical entity, CGH arises from cervical area dysfunctions and pathologies [4].

The characteristic of a TTH is a tight, all-encompassing pain that usually manifests as a single headache with little accompanying symptoms. The most prevalent primary headache is TTH [5]. The second one is the migraine, which is characterized by repetitive episodes of moderate to severe pulsating pain that last anywhere from four to 72 hours. Frequent symptoms include sensitivity toward light, sound, or smell. Usually, during an episode, individuals would rather be still and avoid physical exertion in a quiet, dark place [6]. The origin of pain and its intensity, frequency, and duration differ according to the particular type of CGH. Clinical findings of CGH include a unilateral headache that stays, along with ipsilateral shoulder and cervical pain and stiff neck, which restricts cervical spine freedom of movement. Movement of the neck usually makes the symptoms worse [7]. 

Manual therapy is widely used; in fact recent worldwide survey found that in many countries, it is the most often used complementary or alternative treatment for headache issues [8]. Here, the common interventions include spinal manipulation, spinal mobilization, myofascial release, and other therapeutic massage, although it was widely used after pharmacological intervention in headache management the data was limited [9]. The studies objective is to draw information from recent developments in the use of manual therapy as intervention in headache disorders and to identify the efficacy of the manual intervention on various population. Moreover, the study highlights important areas that should be investigated further to improve clinical practice, education, and healthcare policy in this field.

## Review

The literature search was done on PubMed, MEDLINE, and Google Scholar. Search words included headache disorders or primary headaches or migraine or tension type headaches or cervicogenic headache and manual therapy or spinal manipulation or cranial osteopathy. We searched for all articles published in peer-reviewed journals in English between 2014 and 2024 presenting novel research findings that have been undertaken to explore significant new developments in manual therapies. We considered studies that met the following criteria: RCTs involving individuals aged 18 to 65 years who were identified with migraine [10-13], TTH [14-20], or CGH in accordance with the International Headache Society (IHS) guidelines.

We sought studies that compared therapies such as mobilization, soft tissue, manipulation, cranial osteopathy, or neurodynamic treatments in their intervention. We included research that assessed headaches using any primary evaluation tool. The outcome measures used by various researchers are pain frequency with a headache diary; pain intensity with a visual analog scale, the Headache Index, and the Pain Questionnaire, disability with the Headache Disability Index (HDI), quality of life, cervical range of motion with goniometric measurements, and impact of headache on daily life using the Headache Impact Test (HIT-6). Some studies do not measure outcomes explicitly for headaches; we removed papers where the type of headache (migraine, TTH, or CGH) was linked to a mechanical problem and assessment was focused on them, like shoulder or neck discomfort. Studies assessing pharmaceutical treatments and invasive physiotherapy were also excluded.

Tension headache

Tension headaches are one of the most prevalent types of headaches, often characterized by a dull, aching pain accompanied by a sensation of tightness or pressure across the forehead or on the sides and back of the head. These headaches can vary in intensity from mild to moderate, typically not severe enough to significantly impair daily activities. They can last anywhere from 30 minutes to several hours or even persist for days. The causes are often linked to stress, emotional tension, and anxiety, as well as prolonged muscle tension in the neck, shoulders, and scalp. Poor posture, especially during extended periods of sitting or standing, and eye strain from long hours spent on screens or reading are also common triggers. Effective management usually involves lifestyle changes such as stress reduction techniques, regular physical activity, proper ergonomics, and, in some cases, over-the-counter pain relievers. Understanding and addressing these underlying factors can help mitigate the frequency and severity of tension headaches, significantly improving one's quality of life and review is mentioned in Table 1.

**Table 1:** Review on tension headache

Author/year	Intervention	Duration	Outcome measured	Main findings
Pérez-Llanes et al. [10]	The intervention group received suboccipital muscle inhibition along with the interferential current	20-minute intervention combined with interferential current, applied twice a week for four weeks	Self-reported pain measured using Numerical Pain Rating Scale (NPRS)	The intervention group did not report significantly less pain. Still, they did report less disability and headache impact on daily life, with improvements above the minimum clinically acceptable change
Woodfield et al. [11]	The intervention groups received occiput-atlas manipulation along with soft tissue massage. Control group: Soft tissue massage only	One session for a week for four weeks	Headache disability including subscales for severity, frequency, emotional disability, and functional disability and cervical range of motion (measured by goniometer)	Manipulation was more impactful than massage alone in improving cervical flexion. The manipulation-based treatment was generally more successful than massage alone in lowering the discomfort of headaches on both functional and emotional disabilities
Castien et al. [12]	The intervention consisted of a combination of mobilization of the cervical and thoracic spine, postural correction, and training of isometric strength of neck flexors	Nine sessions of 30 minutes each.	1) Isometric strength of neck flexor duration (seconds), 2) pressure pain scores (PPS, 0-80 points)	Strength of the neck muscles and pain are strongly correlated. Both short- and long-term pain diminishes with increased neck muscle strength, both in the short and long term. Increasing the neck flexor isometric strength lessens pressure discomfort, which is a measure of peripheral or central sensitization in chronic tension headaches
Espí-López [13]	Participants received 1) suboccipital soft tissue inhibition, 2) occiput-atlas-axis manipulation: two-stage manipulation involving cephalic decompression and small circumductions, and 3) combined SI + OAA followed by OAA manipulation	Four sessions over four weeks	Headache Disability Inventory (HDI)	Separately, both techniques alleviated several elements of impairment resulting from tension-type headaches. When compared to the individual treatment and control groups, the combined intervention group had noticeable larger improvements in overall headache disability
Moraska et al. [14]	Participants in the massage group received a 45-minute massage session. The massage consisted of myofascial release and trigger point release	Two times a week for six weeks, for a maximum of 12 sessions	Headache frequency, intensity, and duration	Both massage therapy and placebo treatment reduced headache frequency, although massage resulted in greater perceived clinical improvement and a rise in pain threshold at myofascial trigger points
Ramadan et al. [15]	1st group received instrument soft tissue mobilization, 2nd group received pressure algometry, and 3rd sham ultra sound group.	Two sessions for a four-week session each lasted for 45 minutes	Headache frequency headache disability (by the HURT questionnaire), pressure pain threshold of the upper trapezius and suboccipital muscles, cervical lordosis angle, and anterior head translation	For individuals with tension-type headaches, soft tissue mobilization improved headache symptoms, pressure pain threshold, and cervical alignment better than pressure algometry and sham ultrasound. Comparing to the sham ultrasound group, the pressure algometry group experienced a little drop in headache frequency; however, no additional significant changes were seen among the sham ultrasound and pressure algometry groups
Azhdari et al. [16]	The intervention group received neck muscles manipulation, cranial base release, vertebral mobilization, and trigger points release and suboccipital muscles through friction massage.	3 sessions in 1 week each lasting 30 minutes.	Pain intensity, headache frequency and duration, tablet dosage, and Neck Disability Index	The symptoms of tension-type headaches can be lessened by manual therapy. As study demonstrates decrease in frequency duration of pain and the NDI score and even drug dosage

Migraine

Migraines are a type of headache disorder characterized by intense, throbbing pain, often on one side of the head, accompanied by other symptoms such as nausea, vomiting, and sensitivity to light and sound. Migraines can last from a few hours to several days and can significantly impair daily functioning. They are commonly preceded by warning symptoms known as an aura, which can include visual disturbances, such as flashing lights or blind spots, and other neurological symptoms like tingling in the face or hands. The exact cause of migraines is not fully understood, but they are believed to involve genetic and environmental factors. Triggers can vary widely among individuals but often include hormonal changes (especially in women), certain foods and beverages, stress, sensory stimuli (such as bright lights or loud sounds), changes in sleep patterns, and physical exertion; overall review is mentioned in Table 2.

**Table 2:** Review of literature on migraine

Author/year	Intervention	Duration	Outcome measured	Main findings
Muñoz-Gómez et al. [17]	The intervention group received articulatory techniques. The placebo group received a sham intervention.	One session a week for a period of four weeks.	Intensity and frequency of migraine episodes, migraine disability, quality of life, medication intake, and self-reported perceived change	The articulatory technique-based manual therapy protocol improved physical quality of life and self-observed change post-treatment, and these improvements persisted even after one-month post-intervention. It also decreased in intensity and migraine disability and medication dose. In comparison to the placebo group, the intervention also helped in decreasing the number of migraine episodes and quality of life
Chaibi et al. [18]	The intervention group received chiropractic manipulation. The placebo group received a sham manipulation.	12 sessions over three months, 15 minutes per session.	Number of migraine attacks in a month (days)	The number of migraine days was considerably decreased in all three groups (CSMT, placebo, and control). However, the effect persisted in both intervention and placebo groups during all follow-up periods, while the control group reverted to its baseline. In comparison with the groups receiving a placebo (p = 0.04) and control (p = 0.03), the CSMT group's change in paracetamol usage at the 12-month follow-up was there but considerably smaller
Jiang et al. [19]	Craniosacral therapy (CST) using standardized techniques to free tensions and to balance the cranium and spine.	Twice a week for one hour each time, over four weeks, and four weeks of observation; a total eight weeks duration	Headache Impact Test-6 and headache frequency	The study participants' migraine intensity, frequency, and headache-related disability were all significantly decreased by craniosacral therapy. Anxiety scores and headache-related impairment also decreased in tandem with the reduction in migraine symptoms. No patient experienced any adverse effects during the study indicating the safety of the craniosacral therapy intervention

CGH

CGH is a secondary headache disorder caused by a dysfunction in the cervical spine (neck). This type of headache typically originates from issues with the neck's muscles, joints, nerves, or vertebrae and can result from conditions such as cervical arthritis, disc herniation, or whiplash injuries. Overall review is mentioned in Table 3.

**Table 3:** Review of literature of cervicogenic headache

Author/year	Intervention	Duration	Outcome measured	Main findings
Lerner-Lentz et all. [20]	Both groups received either pragmatically selected mobilization or manipulation techniques applied to the upper cervical spine and a home exercise program given to all subjects	Two sessions in four days	Neck Disability Index, Numeric Pain Rating Scale, and HIT-6	When utilized pragmatically, there was no discernible difference in the results between mobilization and manipulation for patients with cervicogenic headaches. Over time, both groups saw improvements in pain, disability, and headache impact; nevertheless, these improvements were comparable
Ikram et al. [21]	Sustained natural apophyseal glides on cervical spine. Prescribed exercise for other group	Two treatment sessions per week, over eight weeks	Neck Disability Index (NDI), HIT-6, flexion-rotation test and Numeric Pain Rating Scale (NPRS)	While exercises and apophyseal glide mobilization were both useful in treating cervicogenic headache, apophyseal glide approach yielded better outcomes. Compared to the exercise group, the SNAG group experienced considerable reduction in headache intensity, disability, frequency, and duration
Chaibi et al. [22]	Chiropractic spinal manipulation. Placebo group sham manipulation	12 sessions over three months	Number of days with headache per month (primary outcome) and headache duration and intensity improvement	Both the placebo group and chiropractic spinal manipulation group produced long-lasting decreases in the frequency and index of headaches during the follow-up period, while the control group had no change. Although the headache index did not improve until later in the placebo group, the placebo impact was significant, especially when it came to headache frequency. Replication with a bigger sample is required since the inferences that can be taken from the limited sample size are constrained
Mcdevitt et al. [23]	Thoracic spine manipulation and a mobility exercise	Six sessions, with each session last 15 minutes, 1 or 2 per week for up to four weeks	Headache Disability Inventory and Neck Disability Index	While there was no significant improvement in headache-related impairment following thoracic spine manipulation, there were notable reductions in disabilities related to the neck and pain intensity
Nambi et al. [24]	1) Cervical spine manipulation group. 2) Conventional physiotherapy group. 3) Thoracic spine manipulation group	Three times per week for four weeks	Cervicogenic headache frequency (number of painful days)	Up to six months of improvement in headache and neck pain and impairment, as well as days with cervicogenic headache, was observed with manipulation of the cervical spine, which turned out to be more helpful than that of the thoracic spine and traditional physiotherapy. In comparison to the other two groups, the cervical spine manipulation group showed statistically significant improvements in all outcome measures

Since TTHs are the most common type of headache disorder, it have attracted a lot of scientific interest. On the other hand, despite their significant effects, migraine and CGHs have not been thoroughly researched. One explanation for this is that migraines are more difficult to thoroughly study due to their multifactorial nature, which involves intricate genetic, neurological, and environmental components. CGHs, which are secondary headaches caused by cervical spine problems, pose additional research hurdles due to their diverse underlying origins. 

TTH is caused by both peripheral and central sensitization processes, with acute TTH causing greater peripheral excitability and chronic TTH causes central sensitization symptoms. The central nervous system's hypersensitivity may be controlled by manual therapies such as trigger point therapy, joint mobilization, manipulation, and exercise. The effects are mediated by lowering tense muscles and boosting blood flow and oxygenation to the affected areas.

The management of migraines using manual therapy is promising but its more of a complementary therapy as it is mostly helped in reducing the symptoms and decreasing the drug dosage. Although there is a need of more studies to understand the mechanism, few mechanisms are proposed until spinal manipulation therapy which has the potential to activate central descending inhibitory pathways and stimulate neural inhibitory systems. Because it reduces nociceptive input and modifies central pain pathways, its activation may help migraine sufferers feel less pain [25,26].

The craniosacral system consists of membranes, cerebrospinal fluid, and bones that surround the brain and spinal cord. The main idea is that restrictions in the cranial sutures can influence the cerebrospinal fluid's rhythmic impulses, which in turn can affect the brain, spinal cord, and other structures. The goal of craniosacral therapy is to alleviate these limitations in order to return fluid flow to normal. Articulation techniques are believed to promote joint range of motion and stimulate neurophysiological reactions that lead to pain relief. These methods may lessen migraine discomfort and mechanical stress by increasing joint mobility, particularly in the cervical spine and cranial base [27-29].

CGHs are caused by mechanical disturbances or pathological issues to the cervical spine's muscles, ligaments, and nerves. Interventions such as spinal manipulative therapy, mobilization, and soft tissue techniques are designed to address cervical spine dysfunction, reduce nociceptive input, and relieve pain. Effective CGH care also includes measures for reducing central and peripheral sensitization, such as exercise, posture correction, and multimodal pain management [30]. For CGHs, manual and exercise therapy, particularly spinal manipulation, showed modest-to-large impacts in minimizing headache intensity and frequency in the shorter duration, with small-to-moderate effects persisting long-term. Combining different manual therapy techniques appears to be the beneficial strategy for TTHs.

Manual therapy has shown potential for managing several headache conditions. The current evidence, while encouraging, is preliminary and requires more high-quality trials to improve recommendations and establish the efficacy of these therapies and to develop standardized treatment procedures and identify the optimal approaches for each type of headache. Additionally, a interdisciplinary treatment for headaches therapy is required. The subjective nature of headache pain, as well as the potential placebo effect, should be considered, and adequate sham manipulation techniques must be developed to ensure clinical study validity. Furthermore, there is a dearth of details relating the benefits of manual treatment in the younger demographic, where headaches are not insignificant and should not be overlooked as there is only one study found which is trying to develop an assessment tool in children age between seven and 11.

## Conclusions

Osteopathic manipulative treatment (OMT) emerges as a promising complementary approach to the management of headaches. The reviewed studies indicate that OMT can effectively reduce the frequency, intensity, and duration of various types of headaches, particularly TTHs and migraines. Techniques such as myofascial release, cranial osteopathy, and muscle energy techniques are frequently utilized and show favorable outcomes. Integrating OMT into multidisciplinary headache management strategies holds potential for enhancing patient outcomes, improving quality of life, and reducing reliance on pharmacological interventions. This holistic approach aligns with the growing emphasis on patient-centered care and the need for comprehensive pain management solutions.
